# Phosphorylation of Eukaryotic Initiation Factor-2α during Stress and Encystation in *Entamoeba* Species

**DOI:** 10.1371/journal.ppat.1006085

**Published:** 2016-12-08

**Authors:** Holland M. Hendrick, Brenda H. Welter, Matthew A. Hapstack, Steven E. Sykes, William J. Sullivan, Lesly A. Temesvari

**Affiliations:** 1 Department of Biological Sciences, Clemson University Clemson, South Carolina, United States of America; 2 Eukaryotic Pathogens Innovation Center (EPIC) Clemson University Clemson, South Carolina, United States of America; 3 Department of Pharmacology and Toxicology Indiana University School of Medicine Indianaplois, IN United States of America; 4 Department of Microbiology and Immunology Indiana University School of Medicine Indianapolis, IN United States of America; University of Virginia Health System, UNITED STATES

## Abstract

*Entamoeba histolytica* is an enteric pathogen responsible for amoebic dysentery and liver abscess. It alternates between the host-restricted trophozoite form and the infective environmentally-stable cyst stage. Throughout its lifecycle *E*. *histolytica* experiences stress, in part, from host immune pressure. Conversion to cysts is presumed to be a stress-response. In other systems, stress induces phosphorylation of a serine residue on eukaryotic translation initiation factor-2α (eIF2α). This inhibits eIF2α activity resulting in a general decline in protein synthesis. Genomic data reveal that *E*. *histolytica* possesses eIF2α (*Eh*eIF2α) with a conserved phosphorylatable serine at position 59 (Ser^59^). Thus, this pathogen may have the machinery for stress-induced translational control. To test this, we exposed cells to different stress conditions and measured the level of total and phospho-*Eh*eIF2α. Long-term serum starvation, long-term heat shock, and oxidative stress induced an increase in the level of phospho-*Eh*eIF2α, while short-term serum starvation, short-term heat shock, or glucose deprivation did not. Long-term serum starvation also caused a decrease in polyribosome abundance, which is in accordance with the observation that this condition induces phosphorylation of *Eh*eIF2α. We generated transgenic cells that overexpress wildtype *Eh*eIF2α, a non-phosphorylatable variant of eIF2α in which Ser^59^ was mutated to alanine (*Eh*eIF2α-S59A), or a phosphomimetic variant of eIF2α in which Ser^59^ was mutated to aspartic acid (*Eh*eIF2α-S59D). Consistent with the known functions of eIF2α, cells expressing wildtype or *Eh*eIF2α-S59D exhibited increased or decreased translation, respectively. Surprisingly, cells expressing *Eh*eIF2α-S59A also exhibited reduced translation. Cells expressing *Eh*eIF2α-S59D were more resistant to long-term serum starvation underscoring the significance of *Eh*eIF2α phosphorylation in managing stress. Finally, phospho-eIF2α accumulated during encystation in *E*. *invadens*, a model encystation system. Together, these data demonstrate that the eIF2α-dependent stress response system is operational in *Entamoeba* species.

## Author Summary

*Entamoeba histolytica* is the causative agent of amoebic dysentery and liver abscess and is prevalent in underdeveloped countries that lack proper sanitation. Infection is acquired by ingestion of the cyst form in contaminated food or water. During infection, the parasite experiences stress including demanding growth conditions and host immune pressure. Conversion to the infective cyst may be induced by such stress. In other organisms, stress causes a decrease in protein biosynthesis by inducing phosphorylation of eIF2α, which participates in translation initiation. We exposed *E*. *histolytica* to six different stress conditions and observed that some of these conditions (long-term serum starvation, long-term heat shock, and oxidative stress) induced an increase in the level of phospho-eIF2α. Long-term serum starvation was also accompanied by a decrease in mRNA translation. A cell line expressing a mutant version of eIF2α that behaves as a phosphomimetic exhibited decreased translation and increased survival during long-term serum starvation. Finally, phospho-eIF2α accumulated in cysts of *E*. *invadens*, a reptilian pathogen that readily encysts *in vitro*. Together, these data demonstrate that the eIF2α-dependent stress response system is operational in *Entamoeba* and may regulate encystation.

## Introduction

*Entamoeba histolytica* is an intestinal parasite that is the causative agent of amebic dysentery and amoebic liver abscesses. It is transmitted by the cyst form of the pathogen in fecally-contaminated food and water, making it prevalent in the developing world where sanitation practices are substandard. There are 173 million people that live in regions with untreated water sources and one billion people that carry out open defecation practices [1]. Thus, there is considerable risk for transmission of *E*. *histolytica*.

*E*. *histolytica* is passed from human to human without the utilization of an intermediate host. The parasite’s latent stage, a cyst, is able to withstand extreme conditions in the external environment as well as the acidic pH of the host stomach. The cyst exits the stomach and enters the small intestine, where unknown triggers cause excystation. The emerging active trophozoites continue down the digestive system until they reach the large intestine, where they establish infection, feed on bacteria and host cell material, and divide by binary fission. Trophozoites can also invade the colonic epithelial lining and cause extraintestinal complications of infection including liver abscess. During infection the parasite may experience stress, in part, due to immune pressure from the host. This stress can include heat shock, osmotic shock, nutrient deprivation, and/or exposure to reactive oxygen species, nitrogen species, or high oxygen. To survive, the parasite must elicit a cellular response to counter these stresses. *E*. *histolytica* does not readily encyst in axenic culture. Thus, *E*. *invadens*, a related reptilian intestinal parasite that can be induced to encyst *in vitro*, has been widely used as a model system [2,3,4,5,6]. Conversion to latency in *E*. *invadens* is accompanied by increased expression of the heat shock protein, BiP/GRP78 [3]. Thus, encystation is likely to be a stress response in *Entamoeba* species.

In many systems, stress is controlled, in part, by the phosphorylation of the alpha subunit of eukaryotic initiation factor 2 (eIF2) [reviewed in 7]. This factor is a heterotrimeric protein complex consisting of alpha (α), beta (β), and gamma (γ) subunits. In normal growth conditions, eIF2 forms a ternary protein complex with GTP and Met-tRNA_i_. The Met-tRNA_i_ is then delivered to the ribosome to initiate translation. Once Met-tRNA_i_ is delivered, the bound GTP is hydrolyzed to GDP. To become reactivated, eIF2-GDP binds to the guanine exchange factor, eIF2B, and the GDP is released, allowing for the binding of a new GTP. Nucleotide exchange is considered the rate-limiting step of translation initiation [7]. During stress, eIF2 kinases become activated and phosphorylate a key serine residue on the alpha subunit (eIF2α) to generate a phosphorylated form of the protein (phospho-eIF2α). This phosphorylation induces a conformational change in eIF2, causing it to become a competitive inhibitor of eIF2B. This leads to a general decline in protein biosynthesis; however, paradoxically, the expression of a subset of genes is up-regulated. This subset of genes assists the cell in countering stress.

In other eukaryotic pathogens, stage conversion to a latent form is accompanied by phosphorylation of eIF2α. For example, stress induces the parasite, *Toxoplasma gondii*, to convert from a replicating tachyzoite form to a latent bradyzoite form and phosphorylation of eIF2α occurs during this stage transition [8]. Phospho-eIF2α also regulates the formation of latent sporozoites in *Plasmodium* spp. [9] and the transition of promastigotes to amastigotes in *Leishmania* [10]. In non-parasitic organisms, such as yeast [11] and *Dictyostelium* [12], phosphorylation of eIF2α stimulates the formation of latent spores. Genomic data suggest that *E*. *histolytica* and *E*. *invadens* possess the components of this stress-response system [13]. However, the role of eIF2α phosphorylation in the *Entamoeba* stress response has never been characterized.

In this study, we show that phosphorylation of *E*. *histolytica* eIF2α (*Eh*eIF2α) occurs in response to certain stress conditions, namely long-term serum starvation, long-term heat shock, and oxidative stress. Phosphorylation of *Eh*eIF2α is accompanied by a reduction in global translation. We also show that expression of non-phosphorylatable or phosphomimetic forms of *Eh*eIF2α in *E*. *histolytica* influences translation and the ability to counter stress. Finally, we demonstrate that phosphorylation of *E*. *invadens* eIF2α (*Ei*eIF2α) accompanies encystation. Together, these data support the hypothesis that *Entamoeba* species possess an eIF2α-based stress response system that controls protein synthesis, and possibly encystation.

## Results

### *Entamoeba* eIF2α possesses conserved amino acid residues around a key phosphorylated serine residue

**Fig 1:**
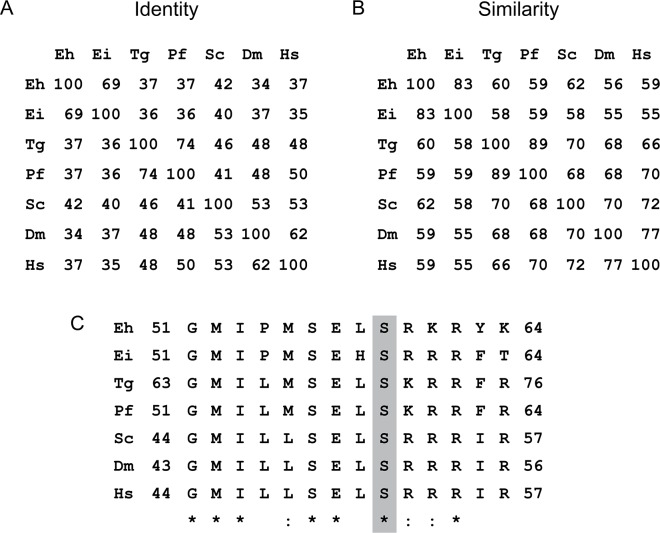
Analysis of eukaryotic translation initiation factor 2α amino acid sequences. Protein identity (A) and similarity (B) matrices were generated using BLOSUM 62 algorithm and Protein Blast. (C) The amino acids around the key serine residue, occurring at position 59 in *E*. *histolytica* were aligned using a Standard Protein BLAST. The key serine residue that becomes phosphorylated is indicated by shading. Fully conserved resides are noted by an asterisk (*) below the residues. Residues showing strongly similar properties are indicated by a colon (:). Amino acid sequences were identified using UniProtKB; UniProtKB accession number identified. Eh, *Entamoeba histolytica* (accession no. C4M0A4); Ei, *E*. *invadens* (accession no. S0AZW3); Tg, *Toxoplasma gondii* (accession no. S8GC56); Pf, *Plasmodium falciparum* (accession no. Q8IBH7); Sc, *Saccharomyces cerevisiae* (accession no. P20459); Dm, *Drosophila melanogaster* (accession no. P41374); Hs, *Homo sapiens* (accession no. P05198)

An alignment of the *E*. *histolytica* eIF2α (*Eh*eIF2α) and *E*. *invadens* eIF2α (*Ei*eIF2α) amino acid sequences with that of five different organisms showed that they shared low sequence identity and moderate sequence similarity across the entire protein with other eIF2α proteins, even when compared to the factor from other eukaryotic pathogens (Fig 1A and 1B). *Eh*eIF2α and *Ei*eIF2α were most similar to each other. Though overall shared homology was low, there was strong sequence identity surrounding the conserved regulatory serine residue, which occurs at amino acid position 59 in the *Entamoebae* (Fig 1C). Thus, serine-59 is likely to be the residue phosphorylated during stress. Conservation around this residue suggests that the machinery for an eIF2α-based stress-response system may be present in *E*. *histolytica* and *E*. *invadens*.

### Stress elicits an increase in the level of phospho-*Eh*eIF2α

**Fig 2:**
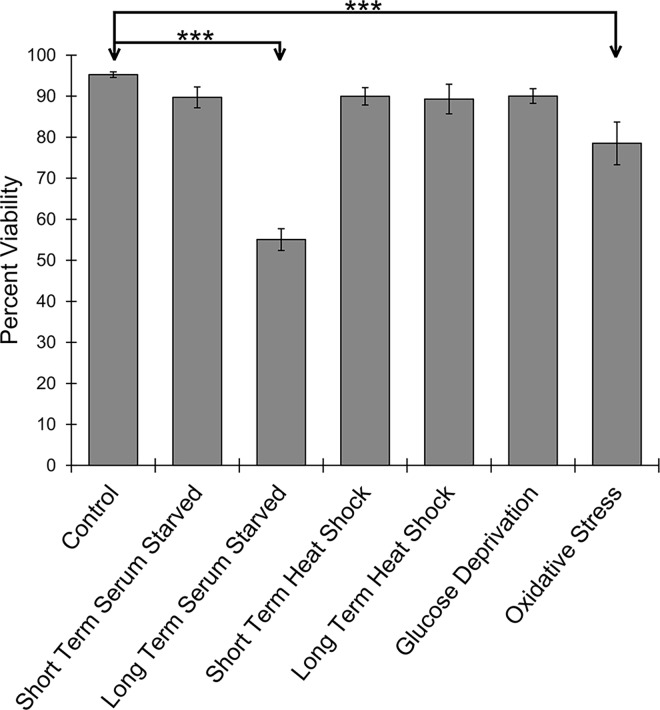
Viability of *E*. *histolytica* trophozoites during stress. Log-phase trophozoites were exposed to a variety of stress conditions as described in the text. Cells were collected by centrifugation and live/dead cells were enumerated via microscopy and Trypan blue exclusion. Percent viable cells is given for each condition. The data represent the mean (± standard error) of at least three separate trials. Significant cell death occurs after long-term serum starvation or oxidative stress (***P<0.001)

To determine if the level of phospho-eIF2α changes during stress, cells were exposed to a variety of stress conditions. Only previously-established stressors were chosen: short- and long-term serum starvation [14], short- and long-term heat shock [15], glucose deprivation [16], and oxidative stress [17]. To confirm that the applied stresses were not inducing significant cell death, which would confound our studies, viability was assessed. Only long-term serum starvation and oxidative stress resulted in statistically significant cell death, albeit the mortality was not complete (Fig 2).

**Fig 3:**
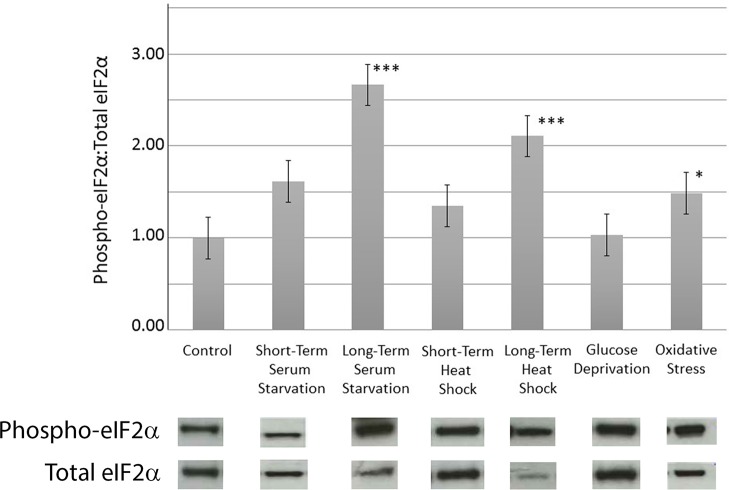
Levels of phospho-eIF2α and total eIF2α levels in control and stressed cells. Control or stressed cells were subjected to Western blot analysis using antibodies specific total eIF2α or phospho-eIF2α. The ratio of phospho-eIF2α to total eIF2α was determined by scanning densitometry and image analysis (Image J, NCBI). The data represent the mean (± standard error) of at least 3 separate trials. All densitometry values were corrected for load using actin and the ratio of phospho-eIF2α to total eIF2α in control unstressed cells was arbitrarily set to 100%. Representative Western blots shown below each stress condition. Only long-term serum starvation, long-term heat shock, and oxidative stress induced a statistically significant increase in the level of phospho-eIF2α (*P<0.05, ***P<0.001). Due to the lability of phospho-eIF2α, it was necessary to perform Western blots for the various conditions at different times. As such, differences in the intensities of bands representing total eIF2α are due to variations in exposure to film from trial to trial.

To track the level of total and phospho-eIF2α during stress, antibodies that specifically recognize the phosphorylated form or total *Entamoeba* eIF2α were generated in rabbits and authenticated by Western blotting (S1 Fig). Western blotting also revealed that there was a basal level of phosphorylated *Eh*eIF2α in control unstressed trophozoites (Fig 3). While all of the stress conditions induced an increase in the level of phospho-*Eh*eIF2α, only trophozoites that experienced long-term serum starvation, long-term heat shock, or oxidative stress exhibited a statistically significant increase in the level of phospho-*Eh*eIF2α (Fig 3). This was not simply due to cell death, since long-term heat shock, which caused minimal cell mortality, induced one of the most dramatic increases in the level of phosphorylated *Eh*eIF2α. These data suggest that an eIF2α-based response system exists in *E*. *histolytica* and is activated in a stress-specific manner.

### Reduced translation accompanies phosphorylation of *Eh*eIF2α

**Fig 4:**
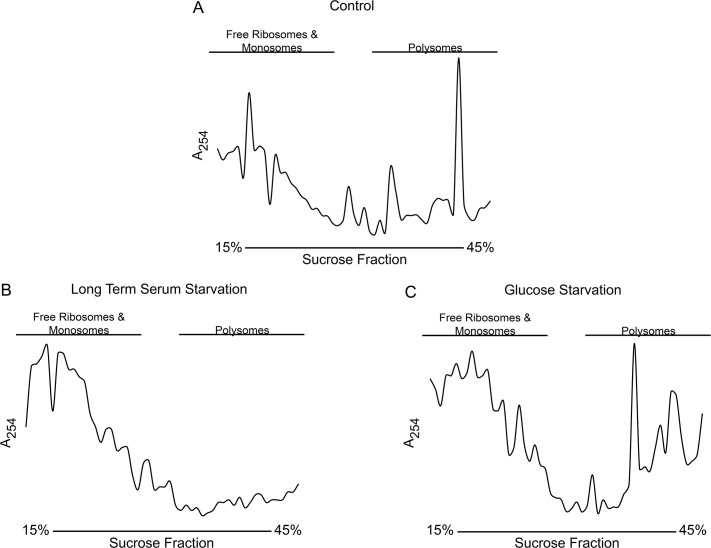
Polyribosome abundance in control and stressed cells. Total RNA from control (A), serum-starved (B), or glucose-starved (C) cells were resolved by sucrose gradient (15–45%) ultracentrifugation, which separates free ribosomes and monosomes (light fractions) from polysomes (dense fractions). The gradients were fractionated and the fractions were analyzed by UV spectrometry (254 nm). Representative profiles of at least 3 separate trials are shown. Long-term serum starvation led to a decrease in large polysome abundance.

In other systems, eIF2α-based control of stress is accompanied by a reduction in global translation [8]. Therefore, we examined global translation in control and stressed *E*. *histolytica* cells by characterizing the abundance of high-density polyribosomes using sucrose gradient ultracentrifugation. We chose two conditions of stress; one that induced phosphorylation of *Eh*eIF2α (i.e., serum starvation), and one that did not induce phosphorylation of *Eh*eIF2α (i.e., glucose deprivation). Consistent with the known function of phospho-eIF2α, serum starvation resulted in a significant reduction in dense polyribosomes and an increase in free ribosomes and monosomes when compared to control cells (Fig 4A and 4B). As expected, glucose deprivation did not result in a decrease in dense polyribosome-bound transcripts (Fig 4C). These results support the premise that *E*. *histolytica* possesses an eIF2α-based stress response system that reduces translation.

### Expression of mutant *Eh*eIF2α alters global translation

To further examine the function of phospho-*Eh*eIF2α, we generated *E*. *histolytica* cell lines that conditionally overexpress mutant forms of *Eh*eIF2α. The cDNA encoding *Eh*eIF2α was mutagenized in two ways using PCR. The codon for serine (S) at position 59 was changed to that of alanine (A) or aspartic acid (D) to produce non-phosphorylatable (*Eh*eIF2α-S59A) or phosphomimetic (*Eh*eIF2α-S59D) forms of *Eh*eIF2α, respectively [18]. Wildtype (unaltered) cDNA was designated *Eh*eIF2α-S59. To distinguish exogenous *Eh*eIF2α from the endogenous form, the 5’ ends of wildtype and mutated cDNAs were also modified to include sequence encoding an N-terminal FLAG epitope peptide sequence (DYKDDDDK) [19] followed by a 5-glycine flexible region.

Modified PCR products were inserted into the *E*. *histolytica* expression vector pGIR209, which confers G418 (neomycin) resistance and allows for tetracycline-inducible expression of exogenous genes [20]. A standard electroporation protocol [21] was utilized to introduce the expression vector into trophozoites that had been previously transfected with another plasmid, pGIR308. This partner plasmid encodes the tetracycline repressor protein, which is necessary for tetracycline inducibility. Authentic transfection was confirmed by purification and sequencing of the episomal expression plasmids from stably transfected cell lines [22]. A previously established cell line that conditionally expresses an irrelevant protein, luciferase, was used as a control [20].

**Fig 5:**
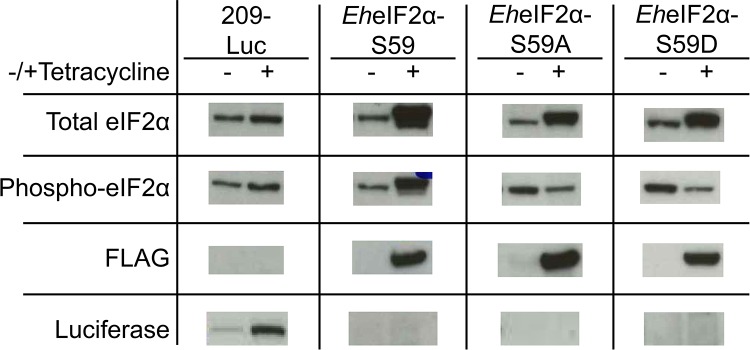
Western blot analysis confirming exogenous protein expression in transgenic cell lines. Trophozoites were transfected with plasmids encoding luciferase (209-Luc), wildtype eIF2α (*Eh*eIF2α-S59), the non-phosphorylatable variant (*Eh*eIF2α-S59A), or the phosphomimetic variant (*Eh*eIF2α-S59D). All exogenous versions of eIF2α were FLAG-tagged at the N-terminus. Protein expression was induced using 5 μg mL^-1^ tetracycline for 24 hours. Western blots of cell lysates were performed using antibodies specific for total *Eh*eIF2α, phospho-*Eh*eIF2α, the FLAG tag, or luciferase. Tetracycline induces expression of exogenous proteins.

Expression of exogenous protein was induced by the addition of 5 μg mL^-1^ tetracycline to the culture medium for a minimum of 24 h. Western blot analysis using anti-FLAG and anti-*Entamoeba* eIF2α showed successful induction of exogenous protein with little to no expression prior to the addition of tetracycline (Fig 5). Interestingly, in the cell line over-expressing the wildtype version of eIF2α, FLAG-tagged *Eh*eIF2α-S59 appeared to be itself heavily phosphorylated. It is not known why exogenously expressed eIF2α is so readily phosphorylated; but, the observation is in accordance with another study in which over-expression of wildtype eIF2α in human A375 melanoma cells resulted in an increase in the basal level of phosphorylated eIF2α [23]. The mutated versions of eIF2α, themselves, cannot be phosphorylated and their expression did not seem to induce an increase in the level of endogenous phospho-eIF2α (Fig 5).

**Fig 6:**
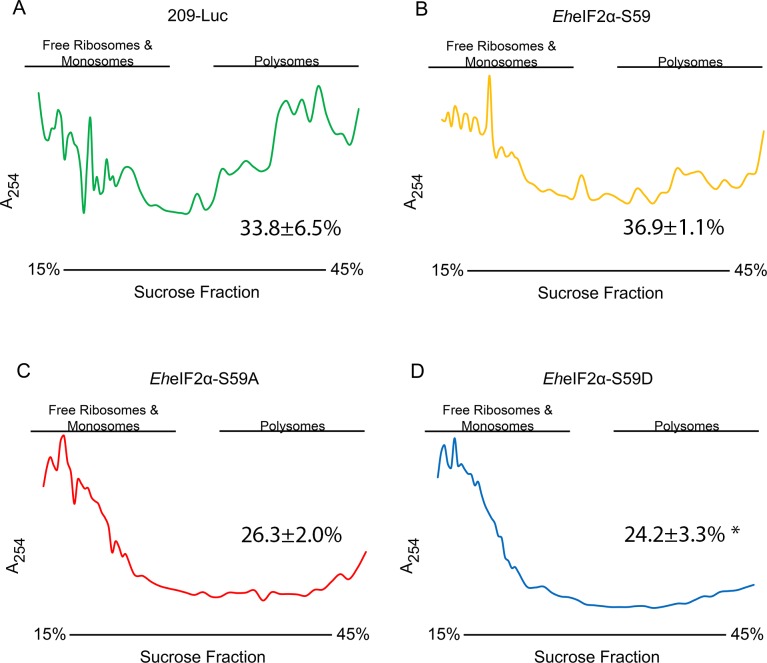
Polyribosome abundance in transgenic cell lines after 72 h of induction of protein synthesis. RNA was isolated from the four transgenic cell lines after incubation in 5 μg mL^-1^ tetracycline for 72 h. The cell lines were the control cell line expressing luciferase, 209-Luc (A), the cell line overexpressing *Eh*eIF2α (B), the cell expressing the non-phosphorylable form of *Eh*eIF2α (C), and the cell line expressing the phosphomimetic form of *Eh*eIF2α (D). The RNA was resolved by sucrose gradient (15–45%) ultracentrifugation, which separates free ribosomes and monosomes (light fractions) from polysomes (dense fractions). The gradients were fractionated and the fractions were analyzed by UV spectrometry (254 nm). Representative profiles of at least three separate trials are shown. The percent of total absorbance in the dense polyribosome fractions (mean ± standard error, n≥ 3) is show in each panel. There is a statistically significant reduction in polyribosome abundance in cells expressing *Eh*eIF2α-S59D (*P<0.05) when compared to control cells (209-Luc).

To confirm that the exogenously expressed *Eh*eIF2α variant proteins were functional, we monitored polyribosome abundance in the transgenic cell lines after 24 or 72 h of tetracycline induction. After 24 h of induction, there was no change in polyribosome abundance in any cell line (S2 Fig). After 72 h of induction, polyribosome abundance remained high in cells expressing luciferase (control) (Fig 6A). This was expected given that expression of this irrelevant protein should not interfere with the translation machinery. Compared to control cells expressing luciferase, there was no statistically significant difference in the abundance of high-density polyribosomes in cells expressing wildtype *Eh*eIF2α-S59 (Fig 6B). However, the polyribosome peaks (Fig 6B) in the *Eh*eIF2α-S59-expressing cells were not as well defined as those in the luciferase-expressing control cell line (Fig 6A). Given that nucleotide exchange on eIF2α represents the rate-limiting step in translation initiation [7], an overabundance of the factor may have relieved this inhibition causing an increase in the translation of mRNAs.

Cells expressing the phosphomimetic variant, *Eh*eIF2α-S59D, exhibited a statistically significant decrease in high-density polysomes after 72 h of tetracycline-induction (Fig 6D). Given that the phosphorylation of eIF2α down-regulates translation, this result was expected. Surprisingly, cells expressing *Eh*eIF2α-S59A also exhibited a decrease in high density polysomes after 72 h of tetracycline-induction, albeit not significantly (Fig 6C). The observation was unforeseen since the presence of unphosphorylated eIF2α is not normally correlated with a decrease in translation. Currently, these unique data cannot be explained, but suggests that the non-phosphorylatable variant of eIF2α is behaving in a dominant negative fashion in *E*. *histolytica* cells.

Since polyribosome profiling provides a snapshot of translation at the mRNA level, we wanted to confirm the alterations in biosynthesis in the mutants at the protein level. Therefore, we used a second method known as SUrface SEnsing of Translation (SUnSET). This non-isotopic technique uses anti-puromycin antibody for the immunological detection of puromycin-labelled proteins [24]. When added to live cell cultures, puromycin, a tyrosoyl-tRNA analog, becomes incorporated into actively translating proteins. Subsequent Western blot analysis of whole cell lysates with anti-puromycin antibody reveals the extent of active protein production in the cell.

**Fig 7:**
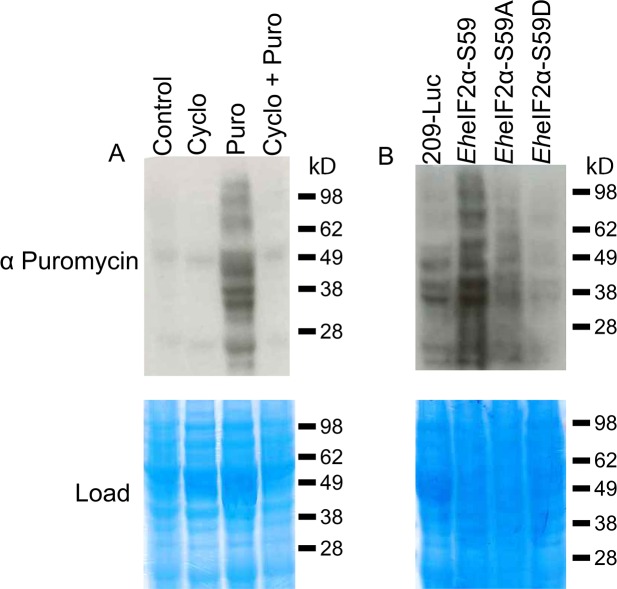
SUnSET analysis of active protein biosynthesis in control and transgenic cell lines. (A) Wildtype cells were incubated in normal growth medium with 100 μg mL^-1^ cycloheximide (Cyclo), 10 μg mL^-1^ puromycin (Puro), or both (Cyclo + Puro). Cell lysates were subjected to SDS-PAGE and Western blotting using antibody specific for puromycin. Trophozoites readily incorporated puromycin into proteins (Puro). The incorporation was authentic since it was blocked by cycloheximide treatment (Cyclo + Puro). This confirms the utility of the SUnSET procedure for assessing protein synthesis in *E*. *histolytica*. (B) Protein expression was induced in the four transgenic cell lines by tetracycline for 72 h and SUnSET was performed. Cell overexpressing the wildtype *Eh*eIF2α showed the highest level of puromycin incorporation, and thus, the highest level of protein synthesis. As expected, cells expressing the phosphomimetic protein (EheIF2α-S59D) exhibited the lowest level of puromycin incorporation in accordance with the known function of phosphorylated versions of eIF2α. Equal protein loads are demonstrated by Coomassie staining of gels (lower panels). Representative data for at least 3 separate trials are shown. Molecular weight (kDa) is shown.

SunSET has been previously used to monitor protein biosynthesis in *E*. *histolytica* [25,26]. To determine if SUnSET could be used to track protein synthesis in *E*. *histolytica* in our hands, wildtype cells were incubated with puromycin before or after incubation with cycloheximide, an inhibitor of protein biosynthesis. Western blotting using anti-puromycin antibody revealed that puromycin was readily incorporated into proteins in *E*. *histolytica* (Fig 7A; “Puro”). The incorporation was specific since there was minimal background staining of lysates from cells that were not treated with puromycin (Fig 7A; “Control”), cells treated only with cycloheximide (Fig 7A; “Cyclo”) or from cells first treated with cycloheximide followed by exposure to puromycin (Fig 7A; “Cyclo + Puro”).

Exogenous protein expression was induced in the transgenic cell lines and then the cells were subjected to SUnSET analysis. Cells overexpressing *Eh*eIF2α-S59 exhibited the highest incorporation of puromycin (Fig 7B) indicating the highest level of protein biosynthesis. In this cell line, increased expression may have been the result of decreased pressure on the rate-limiting step in translation. Consistent with the polyribosome profiles, incorporation of puromycin into proteins in the cell lines expressing *Eh*eIF2α-S59A or *Eh*eIF2α-S59D was low (Fig 7B).

### Expression of mutant *Eh*eIF2α alters viability during stress

**Fig 8:**
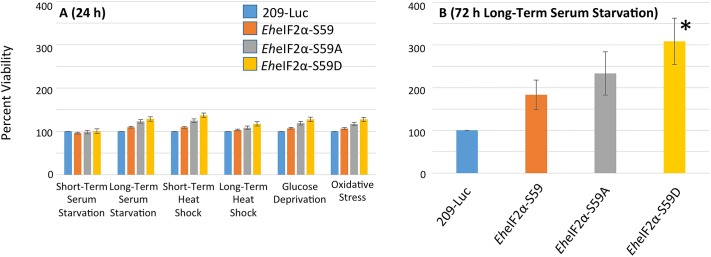
Viability of control and transgenic cell line during stress. (A) Expression of exogenous protein was induced with 5 μg ml^-1^ tetracycline for 24 h after which the cells were exposed to stress as described (see Materials and Methods). Cells were collected by centrifugation and live/dead cells were enumerated via microscopy and Trypan blue exclusion. The cell line expressing *Eh*eIF2αS59D exhibited slightly higher viability in most stresses; however, the increases in viability were not statistically significant. (B) Protein expression was induced in the transgenic cells with 5 μg ml^-1^ tetracycline for 24 h after which the cells were exposed to serum starvation for an additional 48 hours for a total of 72 hours. Although expression of any exogenous protein seemed to afford some protection to long-term serum starvation, the cell line expressing *Eh*eIF2αS59D exhibited a statistically significant increase in viability (*P<0.05). Data represents the mean (± standard error) for at least three separate trials.

The mutant cell lines were then assessed for their ability to survive the various stress conditions applied previously. After 24 h of tetracycline induction followed by application of stress, there was no statistical difference in survivability among the cell lines (Fig 8A). This was not surprising since there was also no detectable defect in protein biosynthesis at 24 hours (S1 Fig). To further explore the ability of the eIF2α variants to offer protection during stress we chose one condition, namely long-term serum starvation, which was able to induce the highest levels of *Eh*eIF2α phosphorylation. Cells were treated with tetracycline for 24 h and then serum-starved in the presence of tetracycline for a total of 72 h. Although, expression of all of the *Eh*eIF2α variants seemed to offer some protection during stress, only cells that expressed *Eh*eIF2α-S59D exhibited statistically significant ability to survive long-term serum starvation. Given that this variant is the phosphomimetic version of eIF2α, it is possible that this cell line was pre-conditioned to handle stress, perhaps by already expressing an important subset of stress-responsive genes.

### Phosphorylation of eIF2α accompanies encystation in *E*. *invadens*

**Fig 9:**
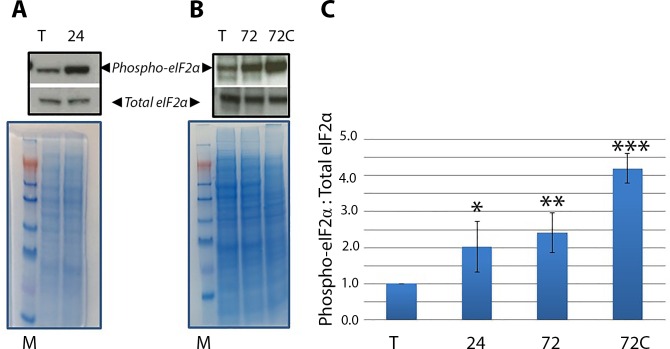
Phospho-eIF2α accumulates during encystation in *E*. *invadens*. (A) Western blot analysis of phospho-eIF2α and total eIF2α in *E*. *invadens* trophozoites (T) or trophozoites induced to encyst for 24 h. (B) Western blot analysis of phospho-eIF2α and total eIF2α in *E*. *invadens* trophozoites (T) induced to encyst for 72 h. The 72 h population was probed before (72) and after (72C) treatment with sarkosyl detergent, which eliminates un-encysted trophozoites from the population. (C) The ratio of phospho-eIF2α to total eIF2α was determined by scanning densitometry and image analysis (Image J, NCBI). The data represent the mean (± standard error) of at least three separate trials. All densitometry values were corrected for load using a single Coomassie stained band and the ratio of phospho-eIF2α to total eIF2α in control unencysted cells was arbitrarily set to 1.0. Representative Western blots are shown. There was significant accumulation of phospho-*Ei*eIF2α in *E*. *invadens* after 24 hours (24, *P<0.05) and 72 hours (72; **P<0.01) into encystation as well as in detergent-purified cysts (72C; ***P<0.001) (B). Coomassie blue staining of SDS-PAGE gels revealed equal protein loading. The lanes with the molecular weight markers (M) are indicated.

Since expression of the conserved 70 kDa heat shock protein (hsp70) increases during encystation [3], stage conversion is presumed to be a stress response in the *Entamoeba* species [3]. Since our antibodies cross-reacted in the *E*. *invadens* system (S1 Fig), the level of total and phospho-*Ei*eIF2α was assessed during encystation by Western blotting. Similar to *E*. *histolytica*, *E*. *invadens* trophozoites possessed a basal level of phospho-eIF2α (Lanes T, Fig 9A and 9B). After 24 h into encystation, there was a significant increase in the level of the phosphorylated form of the factor (Lane 24 in Fig 9A; Fig 9C). The level of phospho-*Ei*eIF2α remained high through 72 h into encystation (Lane 72 in Fig 9B; Fig 9C). It was likely that there were some un-encysted trophozoites remaining in the population at 72 h of encystation. Since detergent-resistance is a hallmark of *E*. *invadens* cysts, we also probed the 72 h population after treatment with sarkosyl. Sarkosyl is a detergent that lyses trophozoites and immature cysts, resulting in a population that consists of detergent-resistant mature cysts. Phospho-*Ei*eIF2α was enriched after removal of detergent-sensitive cells (Lane 72C in Fig 9B; Fig 9C) demonstrating that cysts possess the phosphorylated factor at higher levels than trophozoites. In all cases, equal protein load was measured by Coomassie staining (Fig 9A and 9B). Actin was not used as a load control because actin transcripts are developmentally regulated [5]. Overall, our data show that there is correlation between encystation and the appearance of phospho-*Ei*eIF2α in *E*. *invadens* suggesting that phospho-*Ei*eIF2α plays a role in encystation in the *Entamoeba* species.

## Discussion

This study is the first to demonstrate that stress and encystation can induce phosphorylation of eIF2α in the *Entamoebae*. Specifically, phospho-eIF2α was significantly increased in *E*. *histolytica* as a result of long-term serum starvation, long-term heat shock, or oxidative stress and in *E*. *invadens* after induction of encystation. During long-term serum starvation of *E*. *histolytica*, phosphorylation of eIF2α was accompanied by reduced translation. This suggests that a phospho-eIF2α-based stress response system exists in *E*. *histolytica*. In further support of this, a transgenic cell line expressing a phosphomimetic form of eIF2α exhibited higher viability during long-term serum starvation than cell lines expressing other versions of eIF2α or an irrelevant protein, luciferase. In other words, phosphorylation of eIF2α seems to promote *E*. *histolytica* survival during stress.

Analysis of *E*. *histolytica* genome data suggests that this pathogen possess other components of this stress response system. There are putative homologs for eIF2β (EHI_153480) and eIF2γ (EHI_132880). Furthermore, *E*. *histolytica* possesses two presumptive eIF2α kinases (eIF2K) (EHI_109700, EHI_035950) [13]. Currently, the *E*. *histolytica* eIF2α kinases have not been authenticated, nor have the conditions that lead to their activation been discerned. Nonetheless, the occurrence of each of the three subunits of eIF2, as well as kinases, in genome sequences indicates that this translation factor has a conserved role in this pathogen in delivering Met-tRNAis to the translation machinery. In support of this, cells expressing the phosphomimetic variant of *Eh*eIF2α exhibited reduced polyribosme abundance. As expected, long-term serum starvation, a condition that induces phosphorylation of eIF2α, also exhibited reduced polyribosome abundance.

The ability of the parasite to respond to oxidative stress is critical for virulence functions [reviewed in 27] including the pathogen’s ability to survive in host liver [28]. The observation that eIF2α becomes phosphorylated during oxidative stress in *E*. *histolytica* is in accordance with other studies that demonstrate that phosphorylation of eIF2α protects mammalian cells during oxidative stress [29,30]. Our findings are also consistent with the observation that protein biosynthesis is inhibited during oxidative stress in *E*. *histolytica* [25]. It has been suggested that oxidation of components of the parasite’s translation machinery (e.g., ribosomal proteins, elongation factors) [25], and enzymatic down-regulation of almost all tRNA species [31] are responsible for reduced protein biosynthesis during H_2_O_2_ exposure. Our data show that phosphorylation of eIF2α may also contribute to changes in gene expression and protein biosynthesis during oxidative stress in the pathogen.

It has been reported that serum starvation induces expression of a long non-coding RNA, EhslncRNA [32] and alters ribosome biogenesis by regulating the expression of rRNAs and ribosomal proteins [33]. To date, there have been no genome-wide transcriptomic analyses of serum-starved *E*. *histolytica* cells. Therefore, the identification of the entire set of genes that are down-regulated or up-regulated during this type of stress remains to be seen. However, transcriptomic analyses have been performed on *E*. *histolytica* cells exposed to oxidative stress. Specifically, incubation with H_2_O_2_ induced statistically significant down-regulation of 102 genes and up-regulation of 184 genes [34]. More recently, it has been shown that this differential gene expression may be regulated by a novel transcription factor, which binds to a specific H(2)O(2)-regulatory motif (HRM) in the promoters of genes [35]. Our data suggest that phosphorylation of eIF2α may halt global translation allowing time for the parasite to reconfigure gene expression during exposure to H_2_O_2_.

The eIF2α response to stress appears to be specific to the condition applied since phospho-*Eh*eIF2α did not significantly increase in other presumptive states of stress including short-term serum starvation, short-term heat shock, or glucose deprivation. Interestingly, transcriptomic analyses of *E*. *histolytica* cells exposed to short-term heat shock results in a significant yet general decline in gene expression accompanied by differential expression of the alleles encoding the heavy subunit of the Gal/GalNAc lectin, a parasite surface protein responsible for interaction with host cells [15]. The current study suggests that such short-term heat shock-induced changes in gene expression do not depend on phosphorylation of eIF2α.

Unlike in most glucose-starved mammalian cells [36,37], phospho-*Eh*eIF2α did not accumulate in glucose-starved *E*. *histolytica* trophozoites. While it is accepted that glucose starvation is a “metabolic stressor” in *E*. *histolytica* [16], it is a form of stress that apparently does not induce the expression of the conserved 70 kDa heat shock protein (hsp70) [16]. Instead, glucose starvation in *E*. *histolytica* induces dramatic changes in the expression of other genes and an increase in virulence [16]. Additionally, glucose starvation does not increase the ability of *E*. *histolytica* trophozoites to survive a subsequent challenge such as heat shock or oxidative stress [16]. It is intriguing that a form of stress (i.e., glucose starvation) that does not induce hsp70 expression nor protect cells from subsequent stressors [16] also does not induce phosphorylation of eIF2α (this study).

Given that standard *E*. *histolytica* culture medium is poorly-defined, it is also possible that other carbohydrates serve as a nutrient source in the absence of glucose, which may minimize the stress. This would explain a lack of hsp70 accumulation [16], a lack of phospho-*Eh*eIF2α accumulation (this study), and maintenance of high-density polysomes (this study). *E*. *histolytica* is likely well-adapted to handle a low glucose environment as this would be a common condition in the host large intestine [38]. Our data suggest that the parasite’s response to a low glucose environment does not seem to rely on phosphorylation of eIF2α.

Cells that express the phosphomimetic version of eIF2α exhibited a statistically significant higher ability to survive at least one condition of stress, long-term serum starvation. In general, global translation is reduced by phosphorylation of eIF2α. However, it is known that certain mRNAs escape this inhibition and are selectively expressed even when phospho-eIF2α accumulates. For example, in *Toxoplasma*, 500 gene transcripts are preferentially associated with high-density translation-active polysomes during stress [18]. In mammalian cells, activating transcription factor 4 (ATF4) [39], C/EBP-homologous protein (CHOP) [39], and the α isoform of inhibitor of Bruton's tyrosine kinase (IBTKα) [40] are among those proteins preferentially expressed after phospho-eIF2α accumulates. Interestingly, depletion of IBTKα by short hairpin RNA technology reduces viability of cells during ER stress [40]. Thus, proteins that escape translational control by phospho-eIF2α may be among those key players that determine cell fate during stress. It is possible that in *E*. *histolytica*, expression of the phosphomimetic version of eIF2α, induced preferential expression of proteins, some of which may be protective during stress. This could explain increased viability of trophozoites during long-term serum starvation. Identification of those mRNAs associated with high-density polyribosomes during stress will provide further insight.

In the current study, the expression of wildtype and mutant forms of eIF2α was exogenous. Due to polyploidy, methods for homologous recombination and gene replacement are not yet available for *E*. *histolytica*. As such, in all of the transgenic cell lines, endogenous eIF2α was present. Despite this limitation, we observed phenotypes in the transgenic cell lines. As expected, cells over-expressing wildtype or phosphomimetic variants of eIF2α exhibited increased or decreased translation, respectively. Thus, these exogenous proteins were able to apparently compete with endogenous eIF2α in a predicted fashion.

Surprisingly, in cells expressing the non-phosphorylatable variant, *Eh*eIF2α-S59A, polyribosome abundance and protein biosynthesis were decreased. This phenotype was unexpected because non-phosphorylated eIF2α is generally considered a driver of translation. However, in other systems, the phenotypic outcome of expression of S-to-A variants of eIF2α has not been uniform. For instance, exogenous expression of the non-phosphorylatable variant of eIF2α increases [^35^S]-methionine incorporation into murine 3T3 L1 cells [19]. Overexpression of eIF2α-S51A can transform NIH 3T3 fibroblasts [41] but not 3T3 L1 cells [19]. In systems where gene replacement at both alleles can be achieved, expression of S-to-A variants causes dramatic outcomes including changes in morphology [42], ER stress [42], increases in intracellular reactive oxygen species [29] and cell death [42].

Perhaps *Eh*eIF2α has additional unidentified and non-canonical roles in *E*. *histolytica* and overexpression of the S59A variant leads to a unique phenotype. It might also be possible that the alanine-bearing mutant is behaving in a dominant-negative fashion by titrating other important components of the translation initiation system. Like several other ancient protozoa [43] a canonical ER-based unfolded protein response (UPR) may be incomplete in *E*. *histolytica* [44]. Therefore, the unusual phenotype of the *Eh*eIF2α-S59A-expressing cell line is likely not the result of overexpression of an exogenous protein that leads to ER-stress. In support of this, overexpression of the control protein, luciferase, did not result in a similar decline in polysome abundance (Fig 6A). A thorough examination of the eIF2α binding partners in mutant and control cells will be necessary to fully understand the phenotype.

We demonstrate that the level of phospho-*Ei*eIF2α increases during encystation in *E*. *invadens*. It is well-known that eIF2-based systems are used in eukaryotes for the conversion to latent or dormant forms. Phosphorylation of eIF2α is responsible, in part, for stage conversion in *Toxoplasma gondii* [8], *Plasmodium* spp. [9] *Leishmania* spp. [45], yeast [11], and *Dictyostelium discoideum* [12]. The accumulation of phospho-*Ei*eIF2α during encystation of *E*. *invadens* (this study) is consistent with several previous studies that suggests translation declines during encystation [46,47]. Specifically, the abundance of high-density polyribosomes [46] and the incorporation of exogenous amino acids [47] are decreased in encysting *E*. *invadens* cells. Furthermore, encystation is accompanied by the aggregation of ribosomes into structures known as a chromatoid bodies [47]. Chromatoid bodies are RNA- and ribosomal-containing cytoplasmic granules that arise during stress. They are reminiscent of stress granules that accumulate in an eIF2-dependent manner in other systems [48]. Overall, our data, along with data that show that the levels of stress response proteins, such as hsp70 [3] and hsp90 [49], fluctuate during stage conversion in the *Entamoebae*, support the notion that encystation is a stress response.

Like *E*. *histolytica*, *E*. *invadens* possesses two presumptive eIF2α kinases (EIN_052050; EIN_096010). Expression of the former is developmentally-regulated [5]. Specifically, there is a statistically significant increase in transcript of the former kinase at 24 h of encystation. Interestingly, this surge in transcript level corresponds with the appearance of phospho-eIF2α (Fig 9A). Thus, EIN_052050 is a candidate for regulating the levels of phospho-eIF2α during stage conversion. It remains to be seen if phosphorylation of eIF2α is necessary and/or sufficient to induce stage conversion to the cyst form and further studies with the model organism will provide insight.

In conclusion, we have shown that phospho-eIF2α accumulates in *E*. *histolytica* during a variety of stress conditions and in *E*. *invadens* during encystation. In *E*. *histolytica*, the accumulation of the phosphorylated version correlated with a decrease in translation. To the best of our knowledge, this is the first example of translational control in this pathogen.

## Materials and Methods

### Alignment of eIF2a protein sequences

Amino acid sequences of eIF2α for six model species were aligned individually to *Entamoeba histolytica* eIF2α to examine sequences surrounding the key serine reside. The sequences were also analyzed using a Standard Protein BLAST v 2.3.1 (http://blast.ncbi.nlm.nih.gov/Blast.cgi?PAGE=Proteins) and the BIOSUM 62 algorithm for a “positive” or similarity score, as well as an identity score to form a similarity and an identity matrix.

### Strains and culture condition

*Entamoeba histolytica* (strain HM-1:IMSS) and *Entamoeba invadens* (strain IP-1) were cultured axenically in TYI-S-33 medium in 15 mL glass screw cap tubes at 37°C [50] or 25°C [6], respectively. Cells were passaged into fresh media every 72 to 96 h.

### Induction of stress or encystation

Log-phase *E*. *histolytica* trophozoites were incubated on ice for 10 min to release the cells from the glass surface. Centrifugation was performed at 500 x *g* for 5 min to pellet cells. The cell pellet was resuspended in the appropriate stress medium as follows and incubated at 37°C unless otherwise noted. To induce short-term serum starvation, cells were cultured in TYI-S-33 medium without the addition of adult bovine serum, penicillin-streptomycin, and Diamond’s Vitamins for 1 h prior to analysis. To induce long-term serum starvation, cells were incubated in the same medium for 24 h prior to analysis [14]. Short-term heat shock was induced by incubating trophozoites in complete growth medium in a 42°C water bath for 4 h [15]. To assess long-term heat shock, cells were incubated at 39°C for 24 h. To induce glucose deprivation, trophozoites were incubated for 12 h in TYI-S-33 medium without glucose [16]. To induce oxidative stress, 500 μM hydrogen peroxide (Fisher Scientific) was added to cells in normal TYI-S-33 medium followed by a 45 min incubation [17]. Viability was determined with microscopy using Trypan Blue exclusion (VWR).

Stage conversion (encystation) was induced by incubating *E*. *invadens* trophozoites in 47% LYI-LG, a standard encystation medium with reduced glucose, osmolarity and serum [2,3,4,5,6]. To purify mature detergent-resistant cysts from trophozoites, the 72 h population was incubated in 0.05% (v/v) sarkosyl in PBS at room temperature for 20 min [51].

### Antibody development and western blotting

Antibody development was outsourced (Pierce Biotechnology, Inc., Rockford, IL, USA) and was approved by the Clemson University Institutional Animal Care and Use Committee (Protocol number: AUP2015-021). Briefly, rabbits were immunized with synthetic phosphorylated polypeptide- ILMSEL(pS)KRRFRS and with the unphosphorylated polypeptide EMGTYVALKEYDDIQGMIP targeting phosphorylated or total eIF2α, respectively. These antibodies were purified by ELISA and confirmed against the synthetic polypeptide used in the initial immunization.

SDS-PAGE and Western blot analysis were used to test the specificity of the antibodies and were performed as described previously [52]. Briefly, *E*. *histolytica* or *E*. *invadens* trophozoites (4x10^4^) were collected by centrifugation, resuspended in NuPage LDS buffer (Life Technologies, Carlsbad, CA, USA) and heated for 5 minutes at 95°C. Cell samples were loaded onto a precast 12% Bis-Tris polyacrylamide gel (Life Technologies, Carlsbad, CA, USA). The gel was electrophoresed at 200V and the separated proteins were transferred to a polyvinylidene difluoride membrane (PVDF; Life Technologies) for 1.5 hours at 12V in Towbin buffer. The membrane was blotted with 5% w/v powdered milk in TBST (50 mM Tris, 150 mM NaCl, 0.5% (v/v) Tween 20) for 30 minutes at 37°C. The membranes were incubated overnight (4°C) with rabbit preimmune serum (Day 0) at a dilution of 1:1000 in TBST, serum collected 72 days after inoculation (Day 72) at a dilution of 1:1000 in TBST, or affinity purified antibody at a dilution of 1:500 in TBST. The blots were washed extensively with TBST and incubated for 1 h at 22°C with commercially available horseradish peroxidase-conjugated goat anti-rabbit (dilution factor 1:5000 in TBST) (Fisher Scientific, Fair Lawn, NJ, USA). After washing with TBST, the membrane was developed using the Enhanced ChemiLuminescence Western blotting detection system (Thermo Scientific, Hercules, CA, USA) according to the manufacturer's instructions. Protein was quantified by scanning densitometry (ImageJ, version 1.47, National Institute of Health, USA).

SDS-PAGE and Western blotting of cell lysates generated from stressed *E*. *histolytica* cells or encysting *E*. *invadens* cells were performed as described above. For *E*. *histolytica*-derived samples, a mouse anti-actin commercial antibody was used at a dilution of 1:5000 (Abcam, Cambridge, MA, USA) to confirm equal load. For *E*. *invadens*-derived samples, staining of gels with Bio-Safe Coomassie G-250 Stain (Bio-Rad Laboratories, Hercules, CA) was used to confirm equal load.

### Polyribosome analysis

To halt translation, stressed and unstressed *E*. *histolytica* trophozoites were treated with cycloheximide (100 μg mL^-1^) for 10 min at 37°C. Cells were collected by centrifugation (500 x *g* for 5 min at 4°C). The cell pellet was suspended with cold 1X PBS buffer, washed and resuspended in Breaking/Polysome Buffer (BPB) (10mM Tris-HCl (pH 7.4), 300 mM KCl, 10 mM MgCl2, 1% (v/v) Triton-X-100, 2 mM DTT, 1 mg/ml heparin, 50 μg/ml cycloheximide, and 0.04 units/μl RNase Out) in the presence of protease inhibitors. Lysis was achieved by passing cells twice through a 27-gauge syringe needle. Lysates were clarified by centrifugation at 14,000 × g for 5 min. Samples were loaded onto a 15–45% sucrose gradient in BPB without RNase Out, heparin, DTT or Triton as described previously [53,54]. Ultracentrifugation was performed at 230,000 x g for 2 h. Gradients were fractionated and the fractions were analyzed for polyribosome abundance by spectrophotometry (254 nm) and the absorbance was corrected for cell count.

### Mutagenesis of eIF2α and transfection of *E*. *histolytica* trophozoites

The *E*. *histolytica* eIFα gene is predicted to be intron-less. Therefore, genomic DNA was purified (Wizard Genomic DNA Purification Kit, Promega) and the *Eh*eIF2α gene was isolated by PCR. During PCR, nucleotides encoding a BglII restriction site, a FLAG tag [19], and a 5-glycine flexible region were added to the 5’ end of the gene and nucleotides encoding SalI were added to the 3’ end of the gene.

Site-directed mutagenesis of the codon for serine (S) (TCA; amino acid position 59) to the codon for alanine (A) (GCA) or aspartic acid (D) (GAT) was carried out using a PCR-based protocol using the QuikChange Kit (Stratagene, Santa Clara, CA) according to manufacturer’s instructions. Successful mutagenesis was confirmed by sequencing. Wildtype and mutated *Eh*eIF2α coding sequences were digested with BglII and SalI and ligated into the *E*. *histolytica* expression vector, pGIR209 [20] (gift of Dr. W. A. Petri, University of Virginia, Charlottesville, VA), which had been digested with BglII and SalI. This vector allows for the inducible expression of exogenous proteins via the addition of tetracycline to the medium and is co-transfected with a second vector, pGIR308 [20] (gift of Dr. W. A. Petri, University of Virginia, Charlottesville, VA), which encodes the tetracycline repressor.

Exponentially growing trophozoites of *E*. *histolytica*, harboring pGIR308, were transfected with the engineered pGIR209 vector as described [21]. As a control, amoebae were also transfected with pGIR209 containing the gene encoding luciferase [20]. Transfected amoebae were maintained by adding 6 μg mL^-1^ G418 (pGIR209) and 15 μg mL^-1^ hygromycin (pGIR308) selection agents to the medium.

Expression of exogenous proteins was induced by the addition of 5 μg mL^-1^ tetracycline to the culture medium for 24 to 72 h prior to all studies and confirmed by Western blotting which was performed as described [52] with rabbit anti-luciferase (Invitrogen), anti-FLAG (Sigma), affinity purified anti-eIF2α or affinity purified anti-phospho-eIF2α at dilutions of 1:2500, 1:5000, 1:1000, or 1:1000, respectively. Viability of the mutants during stress and measurements of polyribosome abundance were carried out as described above after 24 h or 72 h post induction of exogenous protein biosynthesis.

### SUrface SEnsing of Translation (SUnSET)

SUnSET analysis has been previously used to assess translational machinery in *E*. *histolytica* [25,26]. To determine if SUnSET could be used to assess protein synthesis in *E*. *histolytica* in our hands, we assessed the incorporation of puromycin into wildtype trophozoites. Cells (2x10^6^) were incubated with 10 μg mL^-1^ puromycin (Sigma-Aldrich) for 15 min before or after incubation with 100 μg mL^-1^ cycloheximide for 10 min. All incubations were held at 37°C. Cells were then pelleted and proteins were precipitated using 20% (v/v) TCA and incubating on ice for 10 min. Proteins were isolated via centrifugation at 2200 x *g* for 5 min and washed with 5% (v/v) TCA. The protein pellet was resuspended in 2X SDS running buffer and incubated in boiling water for 10 min. The lysate was frozen at -80°C until analyzed via Western blot as described above. Mouse anti-puromycin monoclonal antibodies (Sigma-Aldrich) were used at a 1:2500 dilution. As a loading control, samples were stained with Bio-Safe Coomassie G-250 Stain. The protocol was repeated in the transgenic cell lines after a 72 h induction period.

## Statistical analysis

All values are given as means ± standard error of at least 3 trials. To compare means, statistical analyses were performed using GraphPad Prism v.6.05 software with a one-way analysis of variance (ANOVA) and a Tukey-Kramer multiple-comparison test. In all cases, *P* values of less than 0.001 were considered highly statistically significant, while *P* values of less than 0.01 or 0.05 were considered statistically significant.

## Supporting Information

S1 Fig Authentication of antibodies generated to recognize total eIF2α and phospho-eIF2α. Western blot analysis to demonstrate the specificity of anti-total eIF2α (A) and anti-phospho-eIF2α (B) antibodies. In both cases, lysates from 4 X 10^4^ *E*. *histolytica* (*Eh*) or *E*. *invadens* (*Ei*) cells were resolved by SDS-PAGE, transferred to PVDF membrane, and probed with rabbit preimmune serum (Day 0) at a dilution of 1:1000, serum collected 72 days after inoculation (Day 72) at a dilution of 1:1000, or affinity purified antibody (AP) at a dilution of 1:500. Consistent with the known molecular weight of eIF2α (~33.5 kDa), a protein band (red arrow) slightly below the 38 kDa molecular weight marker is visible when Day 72 or affinity purified serum is used as a probe but not when pre-immune serum is used as probe. This demonstrates the specificity of the reagents.
(TIF)

S2 Fig Polyribosome abundance in transgenic cell lines after 24 h of induction of protein synthesis. RNA was isolated from the four transgenic cell line after incubation in 5 μg mL^-1^ tetracycline for 24 h. The cell lines were the control cell line expressing luciferase, 209-Luc (A), the cell line overexpressing *Eh*eIF2α (B), the cell expressing the non-phosphorylatable form of *Eh*eIF2α (C), and the cell line expressing the phosphomimetic form of *Eh*eIF2α (D). The RNA was resolved by sucrose gradient (15–45%) ultracentrifugation, which separates free ribosomes and monosomes (light fractions) from polysomes (dense fractions). The gradients were fractionated and the fractions were analyzed by UV spectrometry (254 nm). Representative profiles of at least three separate trials are shown. After 24 h of induction, there was no change in polyribosome abundance in the transgenic cell lines.
(TIF)
