# Supplementary figures and images for: Phosphorylation of Eukaryotic Initiation Factor-2α during Stress and Encystation in Entamoeba Species
Source: PLoS Pathog. 2016 Dec 8;12(12):e1006085. doi: 10.1371/journal.ppat.1006085 (PMC5179133; doi:10.1371/journal.ppat.1006085)

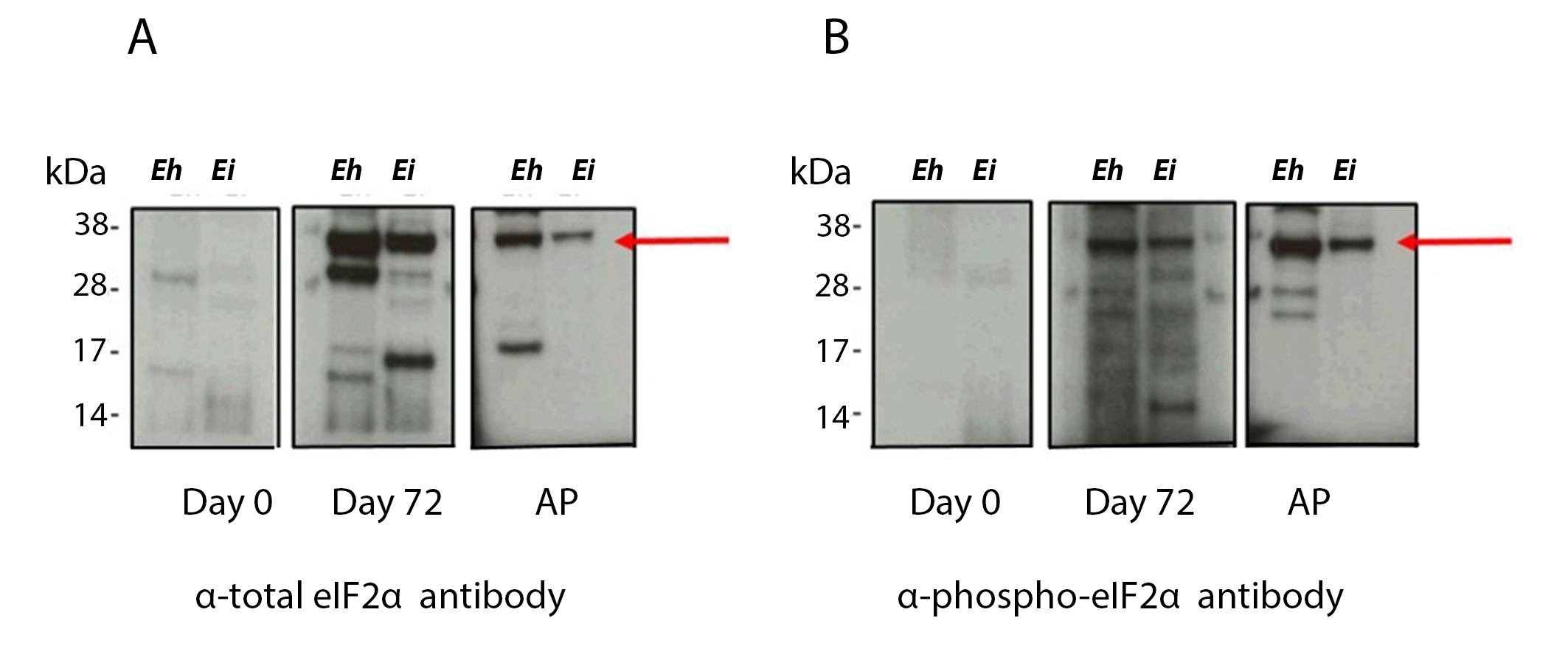

Supplement: S1 Fig — Western blot analysis to demonstrate the specificity of anti-total eIF2α (A) and anti-phospho-eIF2α (B) antibodies. In both cases, lysates from 4 X 104 E. histolytica (Eh) or E. invadens (Ei) cells were resolved by SDS-PAGE, transferred to PVDF membrane, and probed with rabbit preimmune serum (Day 0) at a dilution of 1:1000, serum collected 72 days after inoculation (Day 72) at a dilution of 1:1000, or affinity purified antibody (AP) at a dilution of 1:500. Consistent with the known molecular weight of eIF2α (~33.5 kDa), a protein band (red arrow) slightly below the 38 kDa molecular weight marker is visible when Day 72 or affinity purified serum is used as a probe but not when pre-immune serum is used as probe. This demonstrates the specificity of the reagents. (TIF) [file ppat.1006085.s001.tif]

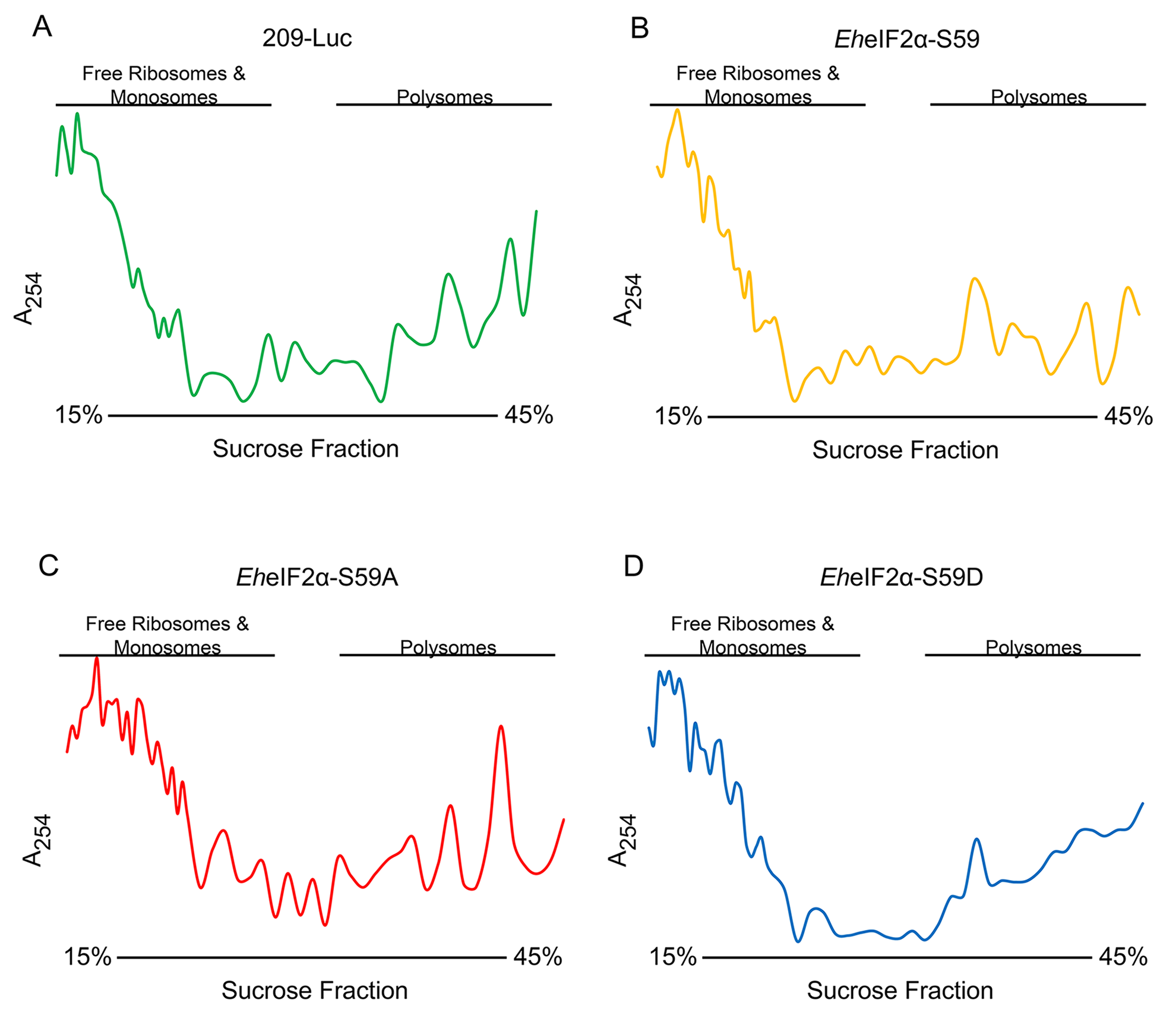

Supplement: S2 Fig — RNA was isolated from the four transgenic cell line after incubation in 5 μg mL-1 tetracycline for 24 h. The cell lines were the control cell line expressing luciferase, 209-Luc (A), the cell line overexpressing EheIF2α (B), the cell expressing the non-phosphorylatable form of EheIF2α (C), and the cell line expressing the phosphomimetic form of EheIF2α (D). The RNA was resolved by sucrose gradient (15–45%) ultracentrifugation, which separates free ribosomes and monosomes (light fractions) from polysomes (dense fractions). The gradients were fractionated and the fractions were analyzed by UV spectrometry (254 nm). Representative profiles of at least three separate trials are shown. After 24 h of induction, there was no change in polyribosome abundance in the transgenic cell lines. (TIF) [file ppat.1006085.s002.tif]
